# Sonographic Diagnosis of an Intercostal Artery Pseudoaneurysm: A Case Report and Literature Review

**DOI:** 10.1002/jcu.70246

**Published:** 2026-04-06

**Authors:** Aaditya P. Singh, Peter R. Coombs

**Affiliations:** ^1^ C/‐ Ultrasound Department Monash Medical Centre Clayton Australia

**Keywords:** fracture, intercostal artery, pseudoaneurysm, sonography, ultrasound

## Abstract

Intercostal artery pseudoaneurysm is a rare complication of blunt trauma or surgical intervention. Haemothorax is the most commonly presenting clinical presentation. We present a case of an intercostal artery pseudoaneurysm diagnosed with ultrasound as an incidental finding in a 63‐year‐old male following a fall. This is a diagnosis mostly made with computed tomography (CT) rather than ultrasound. This case review identifies the role ultrasound can have in this diagnosis.

## Graphical abstract

A 63‐year‐old male presented to the emergency department after being found unconscious during a welfare check. Following a prolonged three‐month hospital admission, the patient developed left flank pain. Spectral Doppler ultrasound revealed a large isoechoic lesion with layering echoes, and spectral Doppler demonstrated bidirectional flow.

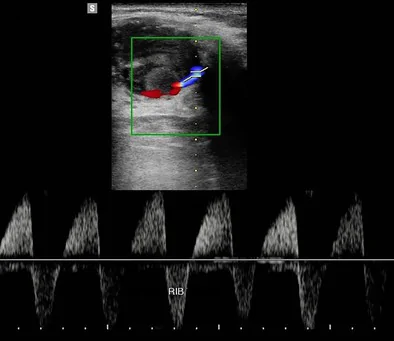

## 1 Background

Intercostal artery pseudoaneurysms (IAP) occur as a complication of chest trauma or surgical intervention. If left untreated, they have an increased risk of rupture that can lead to haemothorax (Atherton and Morgan 1997; Casas et al. 1997; Yamakado et al. 2000; Aoki et al. 2003; Sekino et al. 2005; Kawai and Ito 2009; Long et al. 2012; Chalapathi Rao et al. 2014; Gutierrez Romero et al. 2014; Casper et al. 2019; Liu et al. 2021).

A pseudoaneurysm is a vascular complication that arises because of damage to the arterial wall following iatrogenic or non‐iatrogenic trauma. Trauma to the artery breaches the vessel wall causing an adjacent hematoma. The breach in the wall does not seal and the hematoma becomes encapsulated forming a perfused sac. An arterialized communication between the hematoma and the hole in the artery wall forms commonly referred to as the “neck” (Rivera and Dattilo 2024). This etiology means that a pseudoaneurysm is very different to true aneurysms as pseudoaneurysms do not contain any muscular layer of the artery (Rivera and Dattilo 2024).

IAPs are rare and are generally diagnosed on computed tomography (CT). Angiography is used, in some instances, to follow these up and/or to guide intervention (Jourabchi et al. 2012). There is a paucity of ultrasound reports in the literature (Atherton and Morgan 1997; Casas et al. 1997; Yamakado et al. 2000; Callaway et al. 2000; Bluebond‐Langner et al. 2002; Aoki et al. 2003; Sekino et al. 2005; Takamure et al. 2007; Kawai and Ito 2009; Fernandez Alonso et al. 2009; Long et al. 2012; Jourabchi et al. 2012; Melloni et al. 2012; Chalapathi Rao et al. 2014; Gutierrez Romero et al. 2014; Junck and Utarnachitt 2015; Vajtai and Roy 2015; Casper et al. 2019; Liu et al. 2021; Arnò et al. 2022; Kostopanagiotou et al. 2023). We present a case of an IAP diagnosed as an incidental finding with ultrasound.

## 2 Case

**FIGURE 1:**
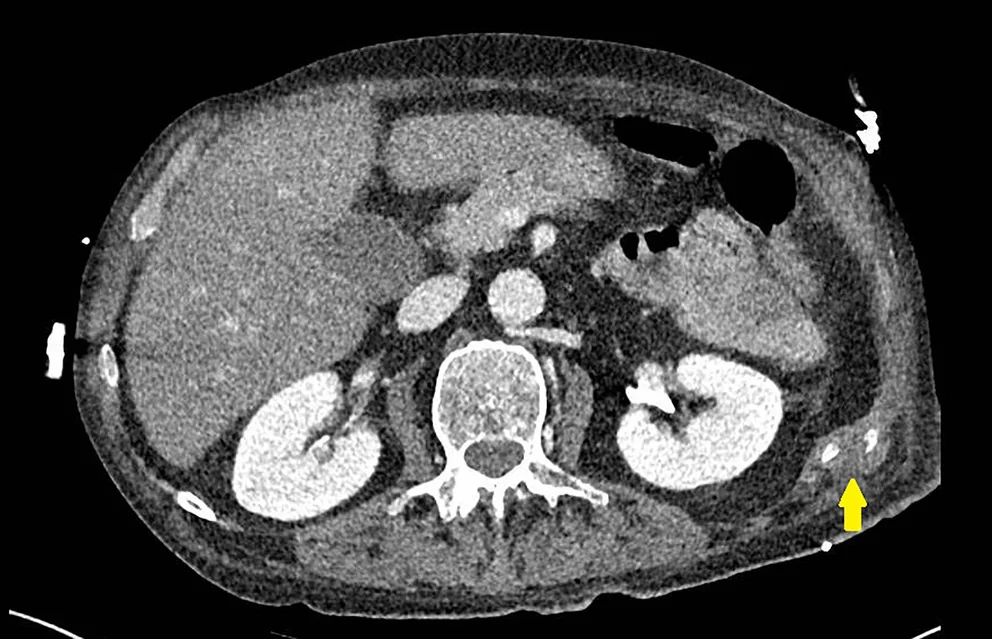
Contrast‐enhanced CT of the abdomen and pelvis in the arterial phase demonstrating a left 11th rib fracture (yellow arrow), obtained 3 days after hospital admission.

**FIGURE 2:**
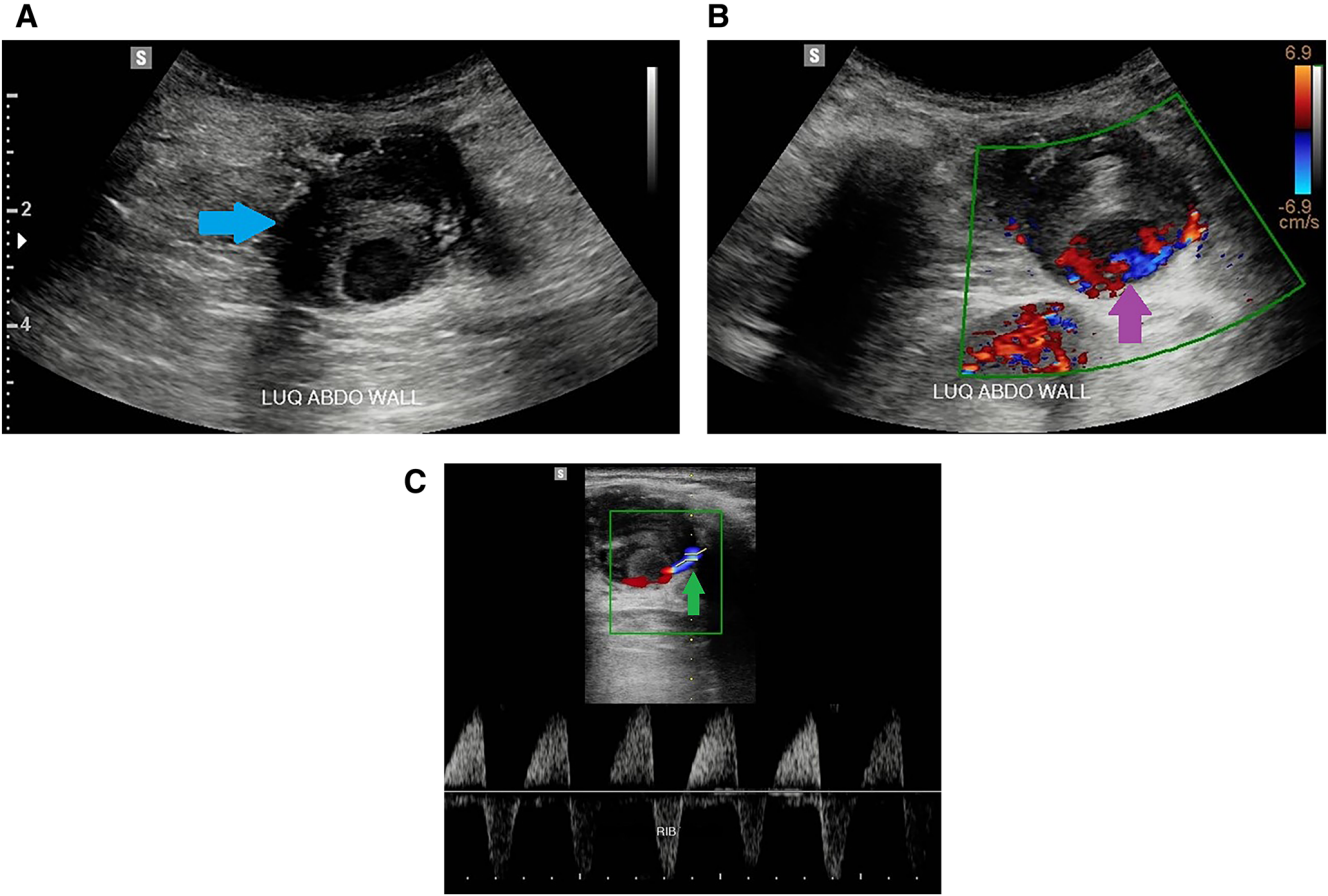
(A) B‐mode ultrasound of the region near the left kidney demonstrating IAP (blue arrow). (B) Color Doppler showing patent flow between the artery and pseudoaneurysm sac with surrounding hematoma (purple arrow). (C) Spectral Doppler confirming bidirectional flow between the sac and artery (green arrow).

**FIGURE 3:**
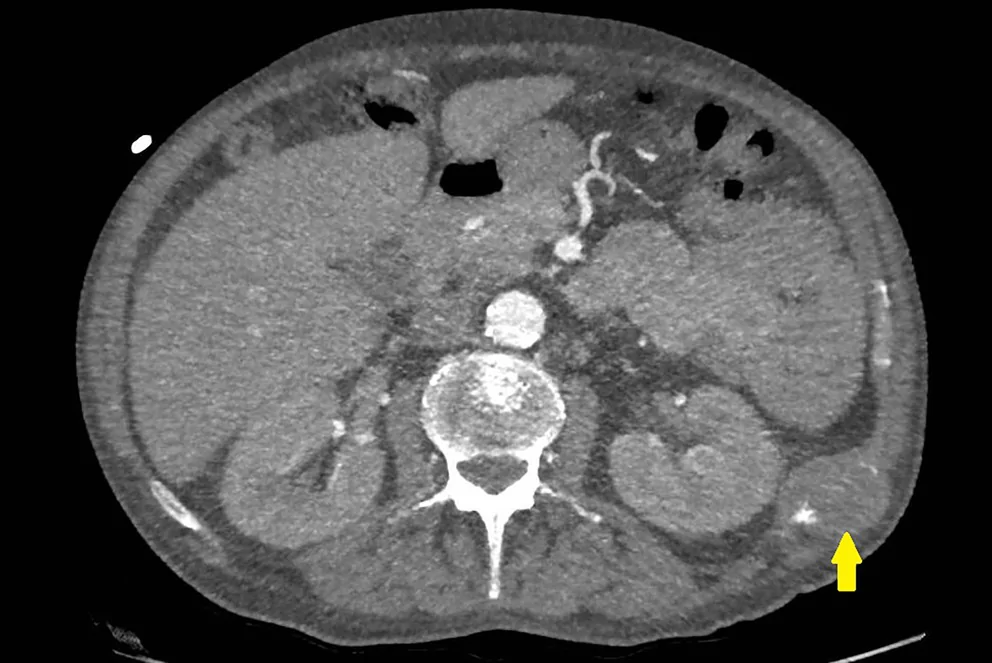
CT intravenous pyelogram (IVP) demonstrating interval change in the size of the 11th rib fracture (yellow arrow), noted 3 months following hospital admission.

**FIGURE 4:**
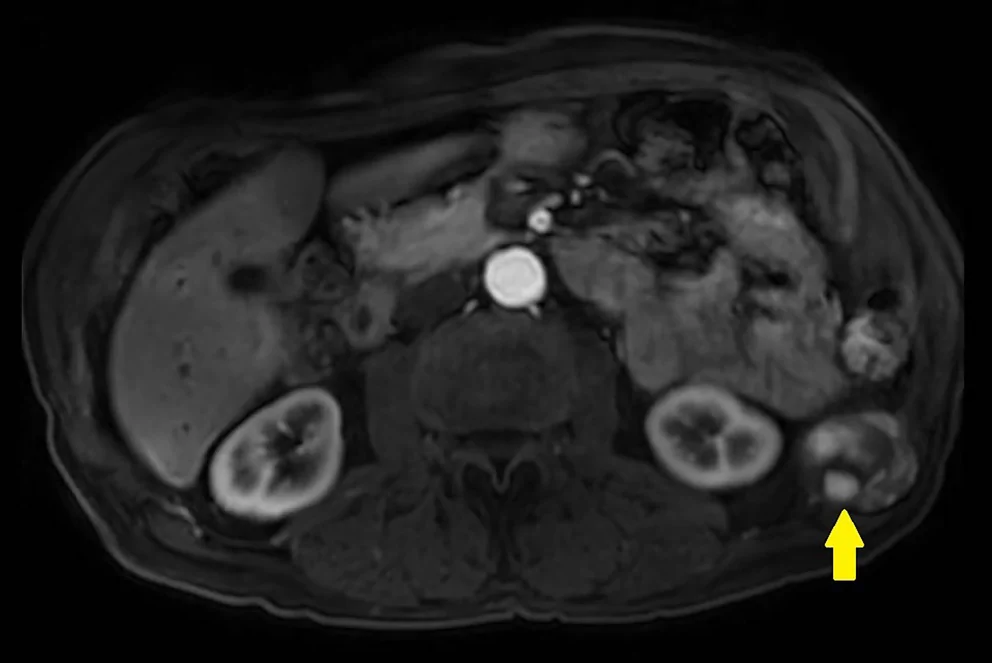
Contrast‐enhanced short‐axis MRI (mDIXON dyn) at 25 s post‐contrast shows persistent enhancement (yellow arrow), indicating sustained arterial perfusion beyond 5 min.

A 63 year—old Caucasian male presented to the emergency department after being found unconscious. CT trauma scan was conducted on the patient which revealed an 11th rib fracture (Figure 1). The patient was hospitalized for treatment and rehabilitation for a lengthy period. Three months later from initial presentation, the patient was diagnosed with Methicillin—Resistant *Staphylococcus aureus* (MRSA) infection. The patient complained of left flank, upper abdomen pain and the doctors were concerned about possible progression of infection into the renal system. An ultrasound was requested to exclude a collection in this region. Ultrasound of the kidney and perinephric region showed no abnormality. Superior and more superficial to the left kidney in the intercostal space was a large isoechoic rounded region measuring 44 × 33 × 42 mm. This region was largely filled with layering echoes presumed to be a chronic hematoma. Approximately a quarter of this region contained swirling echoes on B—mode measuring 18 × 12 × 15 mm (Figure 2a). Color Doppler imaging showed arterialized flow in the region and turbulent flow in a neck between a narrow defect in the artery and the sac (Figure 2b). Spectral Doppler of this neck demonstrated bidirectional flow (Vajtai and Roy 2015) (Figure 2c). This constellation of findings was considered typical of a partially thrombosed pseudoaneurysm. Subsequently, the patient was referred for CT Angiography of the Thoracic region to assess the new incidental finding of IAP. Helical, axial CT acquisition was performed from the inferior neck to the mid abdomen. IV contrast was performed with a delayed arterial phase for 20–30 s. This CT demonstrated heterogeneous, ovoid attenuation within the 11th rib fracture that was suggestive but not definitive for a pseudo‐aneurysm due to motion degraded imaging. There was adjoining mixed density fluid and soft tissue suggesting liquefying hematoma (Figure 3) when compared to the initial CT (Figure 1). Given the limitations of the CT, a multi‐phase magnetic resonance imaging (MRI) was performed to further characterize this region. Subsequently, a multiphase MRI revealed a heterogeneous mass at the site of fracture. The feeding intercostal arteries were evident on the arterial phase imaging and there was a rounded focus of arterial enhancement, consistent with the site of pseudoaneurysm (Figure 4).

**FIGURE 5:**
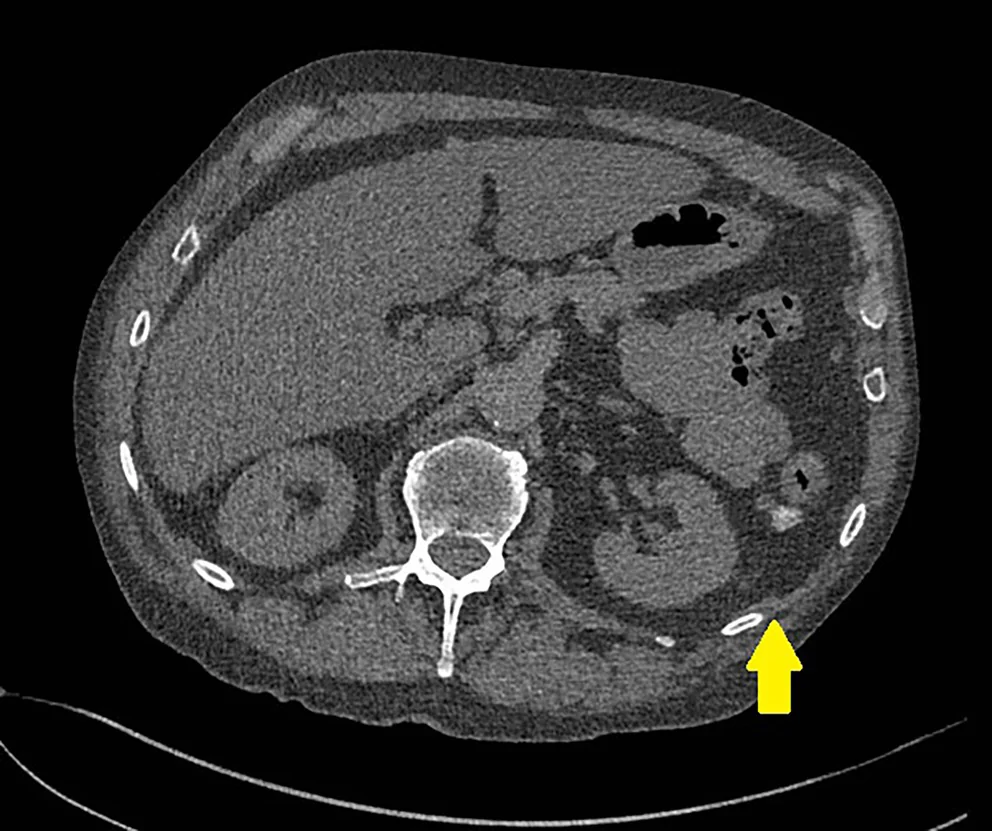
CT of the abdomen and pelvis demonstrating spontaneous resolution of the intra‐arterial pseudoaneurysm (IAP) at the left 11th rib (yellow arrow), 1 year following the initial ultrasound diagnosis.

The diagnosis of an IAP was confirmed. The patient was referred to the interventional radiologist for further monitoring and treatment of the IAP. The interventional radiologist determined that, due to the patient's comorbidities, proceeding with the procedure would be unsafe. As a result, they opted for a conservative management. One year later, a repeat CT demonstrated spontaneous resolution of the IAP (Figure 5).

## 3 Discussion

IAP is extremely uncommon. A narrative literature review of the Pubmed, Ovid Medline, and Scopus databases was performed using combinations of the following search terms. Ultrasound, MRI, angiography, DSA, pseudoaneurysm, false aneurysm, intercostal artery. This strategy identified 28 case reports of IAP (see Table 1).

**TABLE 1:** Literature review of intercostal artery pseudoaneurysm cases by patient characteristics, symptoms, causes, initial diagnosis, and intervention.

Reported cases	Year	Age	Sex	Symptoms	Size	Initial diagnosis (Correlating imaging)	Modality (Intervention)	Causes
Wallace and Nast (1987)	1987	24	Male	Acute chest pain	80 × 120 mm	Chest xray (CT)	Thoracotomy	Aortic coarctation
Atherton and Morgan (1997)	1997	31	Female	Left sided pleuretic chest pain; haemothorax	15 mm	Chest xray (Thoracotomy)	—	Thoracoscopic sympathectomy
Casas et al. (1997)	1997	85	Male	Haemothorax	—	CT (Angiography)	Angiography	Biliary procedure
Yamakado et al. (2000)	2000	62	Male	Haemothorax	—	Angiography	Angiography	Hepatic carcinoma percutaneous microwave coagulation therapy
Callaway et al. (2000)	2000	69	Male	Pulsatile mass	20 mm	Ultrasound (Angiography)	Angiography	Sternotomy wiring
Bluebond‐Langner et al. (2002)	2002	59	Male	Right flank pain	35 mm	CT (Ultrasound)	Spontaneous resolution	Retroperitoneal laparoscopic nephrectomy
Aoki et al. (2003)	2003	68	Male	Haemothorax	15 mm	CT	—	Blunt trauma
Sekino et al. (2005)	2005	36	Male	Haemothorax	20 × 30 mm	CT (Ultrasound)	Angiography	Stab wound
Takamure et al. (2007)	2007	49	Female	—	15 × 10 mm	Autopsy	—	Fell off a bicycle
Kawai and Ito (2009)	2009	66	Female	Haemodynamic instability; haemothorax	10 mm	CT	—	Thoracoscopic lung resection
Fernandez Alonso et al. (2009)	2009	71	Male	Pulsatile mass	21 mm	Ultrasound	Ultrasound	Sternotomy
Long et al. (2012)	2012	63	Male	Haemothorax	8 mm	CT (Angiography)	—	Thoracentesis
Jourabchi et al. (2012)	2012	44	Male	—	20–30 mm	Angiography	Angiography	Irinotecan transarterial chemoembolisation of a spinal metastasis from colorectal cancer
Melloni et al. (2012)	2012	70	Female	Hypertensive crisis	—	CT (Angiography)	Angiography	Computed tomography‐guided percutaneous fine needle aspiration lung biopsy
Lenders et al. (2012)	2012	86	Female	Pulsatile mass	32 × 17 mm	Ultrasound (CT)	Ultrasound	Transcatheter aortic valve implantation
Chalapathi Rao et al. (2014)	2014	32	Male	Haemothorax	—	CT	Angiography	Corrosive acid poisoning
Gutierrez Romero et al. (2014)	2014	66	Female	Haemothorax	—	CT	Angiography	Blunt trauma
Junck and Utarnachitt (2015)	2015	63	Male	Back pain	14 × 12 × 16 mm	CT	Angiography	Spontaneous
Vajtai and Roy (2015)	2015	58	Female	Pulsatile mass	25 mm	Ultrasound	Ultrasound	Ultrasound guided liver biopsy
Agarwal et al. (Agarwal et al. (2016))	2016	47	Female	Acute back pain	—	CT (Angiography)	Conservative Management	Blunt trauma
Philippot et al. (2017)	2017	40	Male	Haemoptysis	—	CT (Angiography)	Angiography	Lobectomy
Wu and Wu (2018)	2018	9	Female	Haemothorax	—	CT (Angiography)	Angiography	Thoracostomy
Casper et al. (2019)	2019	82	Male	Haemothorax	17 mm	CT (Ultrasound)	Angiography	Thoracentesis
Sharma et al. (2019)	2019	34	Female	Haemoptysis	—	CT (Angiography)	Angiography	Pulmonary tuberculosis
Rossi et al. (2019)	2019	82	Male	Pulmonary mass	60 × 55 mm	CT (Ultrasound)	Angiography	Video‐assisted thoracoscopic surgery
Liu et al. (2021)	2021	60	Male	Haemothorax	81 × 75 mm	CT (Angiography)	Angiography	Spontaneous
Arnò et al. (2022)	2022	32	Male	Left Sided Thoracolumbar pain	—	CT (Angiography)	Angiography	—
Kostopanagiotou et al. (2023)	2023	73	Male	Right sided chest pain	40 mm	Xray (CT)	CT	Right lower lobectomy

IAPs appear more commonly in males (18 male, 10 female) and have only been diagnosed in the adult population (ages 9–82). The common clinical presentation is a pulsatile mass or focal pain (Wallace and Nast 1987; Atherton and Morgan 1997; Callaway et al. 2000; Bluebond‐Langner et al. 2002; Fernandez Alonso et al. 2009; Lenders et al. 2012; Junck and Utarnachitt 2015; Vajtai and Roy 2015; Agarwal et al. 2016; Arnò et al. 2022; Kostopanagiotou et al. 2023). They are often accompanied by a hemothorax. The cause of an intercostal artery aneurysm is typically blunt trauma as in our case or surgical intervention. Only two cases report IAPs that occurred spontaneously (Junck and Utarnachitt 2015; Liu et al. 2021). Pseudoaneurysms carry a risk of spontaneous rupture and significant morbidity (Vajtai and Roy 2015). None of the cases in this literature review reported this complication.

A reported method for the treatment of IAP published in the literature is embolization of the IAP (Gutierrez Romero et al. 2014). In some cases, the IAP can spontaneously resolve (Bluebond‐Langner et al. 2002). Further evidence is required to establish whether embolization, thrombin injection, or even direct compression is the preferred intervention when compared to expectant management.

The most common imaging modality used to diagnose intercostal artery aneurysm is CT angiography, which is used for correlating imaging and intervention (Casas et al. 1997; Yamakado et al. 2000; Callaway et al. 2000; Bluebond‐Langner et al. 2002; Aoki et al. 2003; Sekino et al. 2005; Kawai and Ito 2009; Long et al. 2012; Jourabchi et al. 2012; Melloni et al. 2012; Chalapathi Rao et al. 2014; Gutierrez Romero et al. 2014; Junck and Utarnachitt 2015; Casper et al. 2019; Liu et al. 2021; Arnò et al. 2022). Angiography is regarded as the gold standard for the diagnosis of IAP (Casper et al. 2019). None of the reported cases have used MR. While CT, MR, and angiography may be used to diagnose an IAP, each has disadvantages. Angiography has the major drawbacks of cost and that it is a highly invasive procedure. (Vajtai and Roy 2015). CT is less invasive; however, the patients are exposed to ionizing radiation, and some may experience side effects from the contrast medium (Casper et al. 2019). MRI is also costly and invasive.

Ultrasound is the safest method to diagnose intercostal artery aneurysm as ultrasound is non‐invasive and does not expose patients to ionizing radiation. It is also cost effective and readily available. (Fernandez Alonso et al. 2009; Vajtai and Roy 2015). The initial diagnosis of IAPs on ultrasound was reported in only three of the cases (Callaway et al. 2000; Fernandez Alonso et al. 2009; Lenders et al. 2012; Vajtai and Roy 2015). CT is generally preferred in the evaluation of thoracic and abdominal trauma (Gutierrez Romero et al. 2014).

The ultrasound imaging features described are the typical features of an IAP in the literature (Callaway et al. 2000; Sekino et al. 2005; Fernandez Alonso et al. 2009; Vajtai and Roy 2015; Casper et al. 2019) and of a pseudoaneurysm seen in other locations. These include a vascular sac, a communicating neck, and a distinctive bidirectional spectral Doppler waveform (Philippot et al. 2017).

We suspect in our case that an irregular bony margin from the fractured rib penetrated the intercostal artery. This case is novel in a few ways beyond the use of ultrasound to make the initial diagnosis. The IAP in this instance was an incidental finding seen three months after the trauma. Our case did not present with typical symptoms of hemothorax or pulsatile mass; rather, just non‐distinct left‐sided flank pain. This is the only case in the literature demonstrating the role of MR in making this diagnosis.

## 4 Conclusion

IAP is a rare complication of blunt trauma or surgical intervention to the chest. In most cases, hemothorax was the most presenting symptom for intercostal artery pseudoaneurysm. This case demonstrates the contribution ultrasound can make in the initial diagnosis of an intercostal IAP.

## Funding

The authors have nothing to report.

## Ethics Statement

Multiple attempts made to contact the patient however, there are no response. The images and information provided are not identifiable for this case report.

## Conflicts of Interest

The authors declare no conflicts of interest.

## Data Availability

Data sharing not applicable to this article as no datasets were generated or analyzed during the current study.
